# Supplementary material for: Trigonometric function transformation and its application in software reliability modeling
Source: PLoS One. 2025 Dec 31;20(12):e0339286. doi: 10.1371/journal.pone.0339286 (PMC12755828; doi:10.1371/journal.pone.0339286)
Supplement: Attachment — Submitted filename: comments-1.pdf [file pone.0339286.s002.pdf]

Comments to the Author

The authors propose using the trigonometric sine function transformation to model the S-shaped curve of the cumulative number of detection faults during software testing. But the authors did not verify them through experiments.

The problems of this paper are as follows,

1) Although the authors introduced the property of trigonometric sine function transformation, such as $b\sin(t)$ and $b\sin(\beta t+\varphi)+k$, they indeed ignored the limitation of t and $t\geq 0$ during software testing. This restriction will seriously weaken the accuracy of the fault detection rate based on the sine function. I suggest the author compare the fitting and predictive performance of the model proposed in Table 1 with other models. In addition, previous studies have used the sine function to establish software reliability models. I suggest the author to include those models for comparison when conducting experiments.
2) In Subsection 2.2, "Phase 3. The function goes through inflection point, which is the point that the derivation of the function changes from positive to negative quantities." I disagree with this statement. The S-shaped function (i.e. mean value function) in the software reliability model is a non-decreasing function in any situation. In other words, the derivative of the S-shaped function (mean value function) in the software reliability model is never negative under any circumstances.
3) Some grammar errors are as follows,

   Under equations (7,9,11), "Subtitute…" should be "Substitute…", you must check it in the whole paper.

   In the last paragraph of Subsection 3.1, "sin(t) increase…" should be "sin(t) increases…".
