# Supplementary material for: Trigonometric function transformation and its application in software reliability modeling
Source: PLoS One. 2025 Dec 31;20(12):e0339286. doi: 10.1371/journal.pone.0339286 (PMC12755828; doi:10.1371/journal.pone.0339286)
Supplement: Attachment — Submitted filename: Response to Reviewers.pdf [file pone.0339286.s003.pdf]

Firstly, the authors would like to thank the reviewers for their valuable comments. We answer your question/comment as follows:

A. Academic editor

Comment 1:

1. Please ensure that your manuscript meets PLOS ONE's style requirements, including those for file naming. The PLOS ONE style templates can be found at

https://journals.plos.org/plosone/s/file?id=wjVg/PLOSOne_formatting_sample_main_body.pdf and

https://journals.plos.org/plosone/s/file?id=ba62/PLOSOne_formatting_sample_title_authors_affiliations.pdf

Our answer:

We would like to thank you for the comment of the academic editor. We prepare our manuscript using the PLOS ONE style template.

Comment 2:

2. Please note that PLOS One has specific guidelines on code sharing for submissions in which author-generated code underpins the findings in the manuscript. In these cases, we expect all author-generated code to be made available without restrictions upon publication of the work.

Please review our guidelines at https://journals.plos.org/plosone/s/materials-and-software-sharing#loc-sharing-code and ensure that your code is shared in a way that follows best practice and facilitates reproducibility and reuse.

Our answer:

We would like to thank you for the comment of the academic editor. Our manuscript does not have any source code. It provides a theoretical analysis of the application of the transformation of trigonometric functions in Software reliability modelling.

Comment 3:

3. Please update your submission to use the PLOS LaTeX template. The template and more information on our requirements for LaTeX submissions can be found at http://journals.plos.org/plosone/s/latex.

Our answer:

We would like to thank you for the comment of the academic editor. We prepare our manuscript using the PLOS ONE LaTeX template.

Comment 4:

4. Thank you for stating the following financial disclosure:

“This research is supported by Hung Vuong University under grant number HV33.2024”

Please state what role the funders took in the study. If the funders had no role, please state: "The funders had no role in study design, data collection and analysis, decision to publish, or preparation of the manuscript." If this statement is not correct you must amend it as needed.

Please include this amended Role of Funder statement in your cover letter; we will change the online submission form on your behalf.

Our answer:

We would like to thank you for the comment of the academic editor. We have feedback on your comment as follows:

- We move the Role of Funder to the Cover letter as you require.

- Authors Vy Dai-Nghia, Nguyen Van-Thuan, and Nguyen Hung-Cuong are lecturers of Hung Vuong University. This work was done in the lab at our University with the financial support of our University. So we state: "This work was funded by Hung Vuong University (grant HV33.2024)" in the Cover letter.

Comment 5:

5. Please note that funding information should not appear in the Acknowledgments section or other areas of your manuscript. We will only publish funding information present in the Funding Statement section of the online submission form. Please remove any funding-related text from the manuscript.

Our answer:

We would like to thank you for the comment of the academic editor. We will remove the funding information in the Acknowledgments section as you require.

Comment 6:

6. When completing the data availability statement of the submission form, you indicated that you will make your data available on acceptance. We strongly recommend all authors decide on a data sharing plan before acceptance, as the process can be lengthy and hold up publication timelines. Please note that, though access restrictions are acceptable now, your entire data will need to be made freely accessible if your manuscript is accepted for publication. This policy applies to all data except where public deposition would breach compliance with the protocol approved by your research ethics board. If you are unable to adhere to our open data policy, please kindly revise your statement to explain your reasoning and we will seek the editor's input on an exemption. Please be assured that, once you have provided your new statement, the assessment of your exemption will not hold up the peer review process.

Our answer:

We would like to thank you for the comment of the academic editor. Our manuscript does not have any source code. It provides a theoretical analysis of the application of the transformation of trigonometric functions in Software reliability modelling.

Comment 7:

7. PLOS requires an ORCID iD for the corresponding author in Editorial Manager on papers submitted after December 6th, 2016. Please ensure that you have an ORCID iD and that it is validated in Editorial Manager. To do this, go to 'Update my Information' (in the upper left-hand corner of the main menu), and click on the Fetch/Validate link next to the ORCID field. This will take you to the ORCID site and allow you to create a new iD or authenticate a pre-existing iD in Editorial Manager.

Our answer:

We would like to thank you for the comment of the academic editor. I updated my ORCID in My Information in the Editorial Manager system. My ORCID is 0009-0002-5974-8518.

Comment 8:

8. Please include a separate caption for each figure in your manuscript.

Our answer:

We would like to thank you for the comment of the academic editor. We provide a separate caption for each figure in our manuscript.

Comment 9:

9. Please upload a new copy of Figures 1 to 9 as the detail is not clear. Please follow the link for more information: https://journals.plos.org/plosone/s/figures

Our answer:

We would like to thank you for the comment of the academic editor. We update the new version of figures in .tiff and .eps format to improve the quality of figures.

Comment 10:

10. We note you have included a table to which you do not refer in the text of your manuscript. Please ensure that you refer to Table 4 in your text; if accepted, production will need this reference to link the reader to the Table.

Our answer:

We would like to thank you for the comment of the academic editor. It is our mistake. We must refer to Tables 3 and 4 instead of Tables 2 and 3. We update the reference of Table 4 as highlighted on page 10.

Comment 11:

11. If the reviewer comments include a recommendation to cite specific previously published works, please review and evaluate these publications to determine whether they are relevant and should be cited. There is no requirement to cite these works unless the editor has indicated otherwise.

Our answer:

We would like to thank you for the comment of the academic editor. Reviewer 3 mentioned that this work is extended from "Hung-Cuong, N., Quyet-Thang, H., Trung-Nghia, P., Dai-Nghia, V. (2025). Transformating trigonometric function to apply in software reliability modeling. In: Nghia, P.T., Thai, V.D., Thuy, N.T., Huynh, VN., Van Huan, N. (eds) Advances in Information and Communication Technology. ICTA 2024. Lecture Notes in Networks and Systems, vol 1205. Springer, Cham. https://doi.org/10.1007/978-3-031-80943-9_13". So we cite this paper in subsection 3.2 on page 4 to inherit this previous work.

B. Reviewer #1

Comment 1:

Dear authors,

In this study, the transformation of four types of S-shaped functions using trigonometric functions is demonstrated. The mean value function has also been calculated by considering these functions. The idea of using a transformed function in software reliability is novel, but it needs improvements:

1. The literature review is too brief.

Our answer:

We would like to thank you for the comment of reviewer. We add Table 1 on page 4 to list some studied NHPP SRM that use the S-shaped function. Those models provide an overview of previous works.

Comment 2:

2. Writing improvements are needed: the paper contains some lengthy sentences, and paragraphs are not justified. Condensing and improving logical flow is recommended.

Our answer:

We would like to thank you for the comment of reviewer. We have checked the grammatical mistakes in English and edited our manuscript carefully.

Comment 3:

3. It would be better to write the limitations of the transformation, i.e., write the cases when such a transformation is not possible.

Our answer:

We would like to thank you for the comment of reviewer. In our work, the most famous trigonometric function, sin(t), is studied. The domain of this function is the full set of real numbers, i.e., $t \in \mathbb{R}$. So this function can be transformed in all cases.

Comment 4:

4. The representation of Figure 9 may be improved if proper parameters are considered, specially for the red line of b1(t) and orange line of b3(t).

Our answer:

We would like to thank you for the comment of reviewer. We have feedback on your comment as follows:

As mentioned on page 10: "We can see that this form is not fully S-shaped because it only contains the two last phases of the S-shaped curve as described in sub-section 2.2". Furthermore, $b_1(t)$ must pass through the origin of the Cartesian coordinate system, i.e., $t = 0$, then $b_1(t) = 0$. So $b_1(t)$ is illustrated by $b = 2.0$.

- If $\beta < 0$, $b_3(t)$ has the similar shape with $b_1(t)$ which is "*not fully S-shaped*". So $b_3(t)$ is illustrated by $(b, \beta, k) = (2.0, -4.0, 1.5)$.

So we propose to retain this figure to illustrate the S-shape pattern of the 4 types of S-shaped functions.

C. Reviewer #2

Comment 1:

The authors propose using the trigonometric sine function transformation to model the S-shaped curve of the cumulative number of detection faults during software testing. But the authors did not verify them through experiments.

Our answer:

We would like to thank you for the comment of reviewer. Our manuscript provides a theoretical analysis of the application of the transformation of trigonometric functions in Software reliability modelling without any experimental evaluation. The experimental results will be studied and discussed in future works.

Comment 2:

The problems of this paper are as follows,

1) Although the authors introduced the property of trigonometric sine function transformation, such as bsin(t) and b sin(βt + φ) + k, they indeed ignored the limitation of t and t ≥ 0 during software testing. This restriction will seriously weaken the accuracy of the fault detection rate based on the sine function. I suggest the author compare the fitting and predictive performance of the model proposed in Table 1 with other models. In addition, previous studies have used the sine function to establish software reliability models. I suggest the author to include those models for comparison when conducting experiments.

Our answer:

We would like to thank you for the comment of reviewer. We have feedback on your comment as follows:

- In this work, we evaluate the mathematical properties of 4 types of S-shaped functions. All of the previous types of S-shaped function, i.e., $b_1(t)$, $b_2(t)$, and $b_3(t)$, are not considered in the case of $t < 0$.

- Our manuscript provides a theoretical analysis of the application of the transformation of trigonometric functions in Software reliability modelling without any experimental evaluation. The experimental results will be studied and discussed in future works.

- We do not know any SRM that uses the trigonometric sine function. If there is any model that uses the trigonometric sine function, we will use it to compare with our proposed models in our next work.

Comment 3:

2) In Subsection 2.2, "Phase 3. The function goes through inflection point, which is the point that the derivation of the function changes from positive to negative quantities." I disagree with this statement. The S-shaped function (i.e. mean value function) in the software reliability model is a non-decreasing function in any situation. In other words, the derivative of the S-shaped function (mean value function) in the software reliability model is never negative under any circumstances.

Our answer:

We would like to thank you for the comment of reviewer. We agree with your statement: "*The S-shaped function (i.e. mean value function) in the software reliability model is a non-decreasing function in any situation.*". However, subsection 2.2 discusses the value of the derivation of the function. For example, $b_2(t)$ has form:

$$b_2(t) = b\frac{1}{1+\beta e^{-bt}}$$

And the first derivation of the function:

$$\frac{\partial}{\partial t}b_2(t) = \frac{b^2\beta e^{-bt}}{(1+\beta e^{-bt})^2}$$

And in subsection 4.2, we present that the inflection point of $b_2(t)$ is $(\frac{\ln\beta}{b}, \frac{b}{2})$. This means that when $t < \frac{\ln\beta}{b}$, we have $\frac{\partial}{\partial t}b_2(t) > 0$. And vice versa, when $t > \frac{\ln\beta}{b}$, we have $\frac{\partial}{\partial t}b_2(t) < 0$. Furthermore, we change from "*the derivation of the function*" to "*the first derivation of the function*" on page 3 to emphasize our opinion.

Comment 4:

3) Some grammar errors are as follows, Under equations (7,9,11), "Subtitute..." should be "Substitute...", you must check it in the whole paper. In the last paragraph of Subsection 3.1, "sin(t) increase..." should be "sin(t) increases...".

Our answer:

We would like to thank you for the comment of reviewer. We have checked the grammatical mistakes in English and edited our manuscript carefully.

D. Reviewer #3

Comment 1:

This article seems to be an extended study of the following manuscript.

"Hung-Cuong, N., Quyet-Thang, H., Trung-Nghia, P., Dai-Nghia, V. (2025). Transformating trigonometric function to apply in software reliability modeling. In: Nghia, P.T., Thai, V.D., Thuy, N.T., Huynh, VN., Van Huan, N. (eds) Advances in Information and Communication Technology. ICTA 2024. Lecture Notes in Networks and Systems, vol 1205. Springer, Cham. https://doi.org/10.1007/978-3-031-80943-9_13".

Our answer:

We would like to thank you for the comment of reviewer. So we cite this paper in subsection 3.2 on page 4 to inherit this previous work.

Comment 2:

The novel contribution of this paper is limited.

Our answer:

We would like to thank you for the comment of reviewer. We have feedback on your comment as follows. In abstract, we address our contribution in this manuscript as follows: "*The first contribution is a deep mathematical appreciation of three well-known S-shaped functions. The second is the mathematical transformation of the trigonometric function to meet the real assumption. The last are the advantages and the applicability of this transformation in software reliability modeling.*". All of those contributions have not been studied in any previous works.

Comment 3:

This paper contains many grammatical mistakes.

English writing need to be improved, severely.

Our answer:

We would like to thank you for the comment of reviewer. We have checked the grammatical mistakes in English and edited our manuscript carefully.
