# Supplementary material for: Trigonometric function transformation and its application in software reliability modeling
Source: PLoS One. 2025 Dec 31;20(12):e0339286. doi: 10.1371/journal.pone.0339286 (PMC12755828; doi:10.1371/journal.pone.0339286)
Supplement: Attachment — Submitted filename: Review report.docx [file pone.0339286.s001.docx]

Comment to the authors of “Trigonometric function transformation and its application in software reliability modelling (PONE-D-25-53209)”

Dear authors,

In this study, the transformation of four types of S-shaped functions using trigonometric functions is demonstrated. The mean value function has also been calculated by considering these functions. The idea of using a transformed function in software reliability is novel, but it needs improvements:

1. The literature review is too brief.
2. Writing improvements are needed: the paper contains some lengthy sentences, and paragraphs are not justified. Condensing and improving logical flow is recommended.
3. It would be better to write the limitations of the transformation, i.e., write the cases when such a transformation is not possible.
4. The representation of Figure 9 may be improved if proper parameters are considered, specially for the red line of b1(t) and orange line of b3(t).
