# Supplementary material for: Trigonometric function transformation and its application in software reliability modeling
Source: PLoS One. 2025 Dec 31;20(12):e0339286. doi: 10.1371/journal.pone.0339286 (PMC12755828; doi:10.1371/journal.pone.0339286)
Supplement: Attachment — Submitted filename: Review report R1.docx [file pone.0339286.s004.docx]

Comment to the authors of “Trigonometric function transformation and its application in software reliability modelling (PONE-D-25-53209_R1)”

Dear authors,

Since the authors answered all my queries, the article may be accepted for publication after checking the format.
